# Liquid-Phase Approach to Glass-Microfiber-Reinforced Sulfide Solid Electrolytes for All-Solid-State Batteries

**DOI:** 10.1021/acsami.3c01383

**Published:** 2023-07-19

**Authors:** Hany El-Shinawi, Ed Darnbrough, Johann Perera, Innes McClelland, David E. J. Armstrong, Edmund J. Cussen, Serena A. Cussen

**Affiliations:** † Department of Materials Science and Engineering, University of Sheffield, Mappin Street, Sheffield City Centre, Sheffield S1 3JD, United Kingdom; ‡ Chemistry Department, Faculty of Science, Mansoura University, Mansoura 35516, Egypt; § Department of Materials, University of Oxford, Parks Road, Oxford OX1 3PH, United Kingdom; ∥ The Faraday Institution, Quad One, Harwell Science and Innovation Campus, Didcot OX11 0RA, United Kingdom

**Keywords:** solid-state batteries, solid electrolytes, sulfide electrolytes, thin composites, glass microfiber

## Abstract

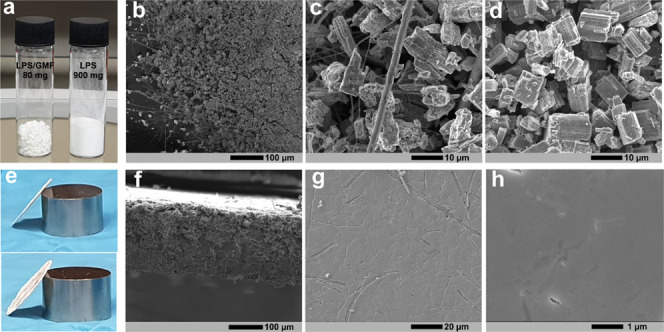

Deformable, fast-ion conducting sulfides enable the construction of bulk-type solid-state batteries that can compete with current Li-ion batteries in terms of energy density and scalability. One approach to optimizing the energy density of these cells is to minimize the size of the electrolyte layer by integrating the solid electrolyte in thin membranes. However, additive-free thin membranes, as well as many membranes based on preprepared scaffolds, are difficult to prepare or integrate in solid cells on a large scale. Here, we propose a scalable solution-based approach to produce bulk-type glass-microfiber-reinforced composites that restore the deformability of sulfide electrolytes and can easily be shaped into thin membranes by cold pressing. This approach supports both the ease of preparation and enhancement of the energy density of sulfide-based solid-state batteries.

## Introduction

All-solid-state batteries comprising solid-state electrolytes allow for the development of high-energy-density batteries that operate safely over a wide range of temperatures.^1,2^ Sulfide-based solid electrolytes have shown themselves to be an excellent prospect for lithium-based batteries.^3−5^ They display excellent Li-ion conductivities, exceeding some liquid organic electrolytes, and can be easily prepared as continuous bulk solid electrolytes by pressing the material at room temperatures.^6^ This facilitates the construction of an all-solid-state battery when combined with electrode materials after applying the appropriate coatings.^7^ To maximize energy density, there is a drive to minimize the amount of the solid electrolyte while maintaining electronic insulation and sustaining Li-ion conduction pathways.^8,9^ Several approaches have been recently suggested to prepare thin sulfide-based solid electrolyte membranes, including dry-mixing of the solid electrolyte with a binder such as polyimine followed by hot pressing,^10^ tape casting a slurry of the solid electrolyte followed by cold pressing (with or without the application of a reinforcing scaffold),^11,12^ or infiltrating a slurry of the solid electrolyte in preprepared scaffold matrices.^13,14^ However, the main downside of these procedures, apart from the complexity of some of these techniques, is their reliance on preparative techniques, which may be challenging to scale up, e.g., high-energy ball milling and/or high-temperature treatments in quartz ampoules.^3,4^

Liquid-phase reactions have recently been suggested as an alternative preparative method for sulfide-type solid electrolytes.^15−19^ These syntheses are often performed at lower temperatures (<300 °C), offering several advantages over solid-state synthesis in terms of synthesis temperature, synthesis time, and scalability.^17^ Moreover, they can lead to the stabilization of metastable fast-ion conducting phases such as β-Li_3_PS_4_ and Li_7_P_3_S_11_.^15,16,18^ Recently, liquid-phase synthesis has been used in evaporation-induced self-assembly to produce additive-free ultrathin sulfide solid electrolyte membranes.^20,21^ However, integrating these thin electrolytes into high-energy cells is challenging because the thin electrolyte layer is fragile and breaks easily during cell fabrication or operation against Li anodes. In this paper, we report a simple and scalable approach to preparing thin sulfide-based solid electrolyte membranes based on liquid-phase synthesis. This modified approach directly produces bulk-type β-Li_3_PS_4_/glass-microfiber (LPS/GMF) composites, which are robust to handling and easily shaped in thin membranes for use in all-solid-state batteries.

## Experimental Section

### Synthesis and Cell Assembly

LPS/GMF composites (15 wt % GMF) were synthesized using a modified liquid-phase approach. Stoichiometric amounts of Li_2_S and P_2_S_5_ (Sigma-Aldrich) were reacted in tetrahydrofuran (THF) overnight to produce a Li_3_PS_4_·3THF precursor;^16^ in a separate container, GMF (Whatman; Sigma-Aldrich) was dispersed in THF via sonication. The THF solutions containing Li_3_PS_4_·3THF and GMF were then mixed and stirred for 3 h, and then the THF solvent was removed by centrifugation. The resulting solid precursor was then dried under vacuum before heating at 145 °C to completely remove THF and fully crystallize β-Li_3_PS_4_. To investigate the influence of GMF on the solid electrolyte performance, single-phase LPS was also synthesized from the same procedure. For ionic conductivity measurements, LPS/GMF composite (18 mg) and LPS (100 mg) were pressed separately between two carbon-coated Al foils using a 10 mm die at 250 MPa. To prepare a symmetric Li/LPS-GMF/Li cell, two small pieces of Li foil (∼2 mg each) were first separately pressed on top of thin Ni foils (8 mm in diameter) at 156 MPa. These Li/Ni discs were then attached to both sides of a prepressed thin LPS/GMF disc (22 mg mass of composite; 10 mm dies; 250 MPa). To prepare a full Li/LPS-GMF/LTP solid-state cell, the nanostructured LiTi_2_(PO_4_)_3_/C (LTP/C) was prepared according to the published procedure.^22^ LTP/C powder was then dispersed in THF, drop-cast onto a thin Al foil (8 mm in diameter), and dried under vacuum at 120 °C. LTP/C and Li/Ni discs were then attached to opposite sides of a prepressed thin LPS/GMF disc; the three cell components were finally pressed at 4900 N to construct the solid-state battery in a Swagelok cell.

### Characterization

X-ray diffraction (XRD) analysis was performed using a Rigaku MiniFlex diffractometer in reflection mode using Cu Kα radiation. The powder materials were sealed in an airtight sample holder in an argon-filled glovebox to prevent reaction with moisture. Scanning electron microscopy (SEM) was performed using an FEI Inspect F50 scanning electron microscope, using a nominal acceleration voltage of 10 kV. Energy-dispersive X-ray (EDX) was collected using an Oxford Instruments Aztec Energy EDS Analysis System. Samples for SEM/EDX analyses were prepared in an argon-filled glovebox. Impedance spectroscopy and cycling tests of the assembled cells were performed using a BioLogic VMP300 potentiostat. Impedance measurements were performed in the frequency range of 1 Hz to 5 MHz with an applied voltage of 0.05 V.

Indentation tests were undertaken on samples prepared as outlined previously and mounted onto an SEM stub, as is the standard practice for nanoindentation.^23^ The Hysitron PI88 in situ nanoindentation equipment was then used to conduct 7–11 indents using the CMX method in 5 distinct locations of a sample with or without GMF. This produces storage modulus and hardness results with depth.^24^ The mean modulus/hardness data for indents in each location is taken to produce a plot of modulus/hardness vs depth. The mode of this data is then taken as the expected value for a location with the standard error calculated from the variance and the number of tests conducted in each area.

For bending tests, samples were cut with a scalpel from circular pellets to give approximately parallel-sided strips, which were then mounted at one end using superglue to form a macroscale cantilever. These samples were then tested at increasing lengths using a Hysitron PI88 in situ nanoindentation system. These tests recorded the force (*F*) and displacement (*y*) of the indenter tip. To find the elastic response of the cantilevers, the gradient of the unloading force–displacement curve was fitted linearly to give a value, which is dependent on the flexural modulus, the length of the cantilever tested, and the second moment of area (eq 1). Solving eq 1 using the standard Bernoulli–Euler beam theory, knowing the dimensions of the specimen gives a flexural modulus. All dimension measurements were taken from SEM images
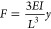
1The flexural modulus assumes that the elastic modulus is uniform throughout and equal in compression and tension. If you disregard the second assumption, you can see that the standard beam theory can be solved based on the axial stress being zero and the bending moment in compression and tension being equal to give a relationship between the apparent flexural modulus with the compressive modulus and tensile modulus (eq 2). The values from beam bending are used as *E*_flexural_ and the nanoindentation values as compression *E*_c_(25)
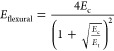
2
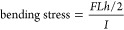
3where *F* is force, *L* is the length of the cantilever tested, *h* is height, and *I* is the second moment of inertia.

## Results and Discussion

**Figure 1:**
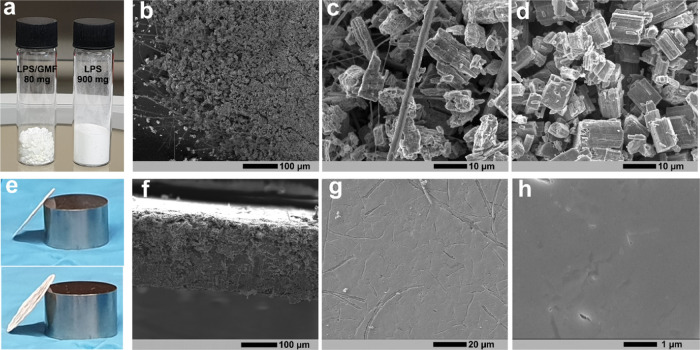
(a) Photograph of as-synthesized LPS/GMF and GMF-free LPS powders (80 and 900 mg mass, respectively); (b, c) SEM images of LPS/GMF; (d) SEM image of single-phase LPS; (e) photographs of a thin LPS/GMF pellet (∼20 mg of composite material pressed at 125 MPa using 10 mm dies; the metal cylinder in the photograph is the 10 mm die used in pressing); (f) SEM image showing a cross section of the LPS/GMF pellet; and (g, h) surface SEM images of the LPS/GMF pellet.

To choose the optimum weight content of GMF, LPS/GMF composites of 10, 15, and 20 wt % GMF were prepared. The composite with a 10 wt % GMF displayed difficulty in processability, as thin pellets were too fragile to handle and accurately characterize. At 15 wt % of GMF, handling was noticeably improved. A significant drop-off in conductivity was found for the 20 wt % GMF (Figure S1); the 15 wt % composition was thus selected for further investigation. The texture and microstructure of the synthesized LPS/GMF composite (15 wt % GMF) are presented in Figure 1. The incorporation of GMFs visibly decreases the tap density, as shown in Figure 1a, compared with single-phase GMF-free LPS. SEM images of the two materials (Figure 1b–d) reveal that the LPS in the GMF composite retained the nanostructured porous morphology, which is characteristic of liquid-phase-synthesized LPS.^16^

XRD patterns collected from the LPS/GMF composite and single-phase LPS (Figure S2) indicate no effect of GMF incorporation on the LPS phase purity or structure. The low apparent mass/volume ratio of the LPS/GMF composite (Figure 1a) enables the ease of handling and processing of small quantities of material in the form of membranes of controllable thicknesses by cold pressing known quantities of the material in a given die. This allowed for the production of thin membranes down to 100 μm in thickness (Figure 1e,f). Attempts to produce membranes of similar thicknesses of single-phase, GMF-free LPS led to pellet breakage under pressing, with the resulting fragments being too fragile to handle. Figure 1f–h shows SEM images of 20 mm diameter LPS/GMF membranes cold-pressed at 125 MPa. These images reveal a bulk material, where the LPS is transformed from individual particles to one continuous self-supporting medium. This is in contrast to the surface of a single-phase LPS pellet where individual particles are still visible (Figure S3). This behavior is attributed to the GMF scaffold, which is stiffer than LPS; under compression, the LPS particles can deform around the GMFs filling in any gaps. In single-phase LPS, the stress is evenly distributed over all particles leading to less deformation and subsequent retention of gaps between particles.

**Figure 2:**
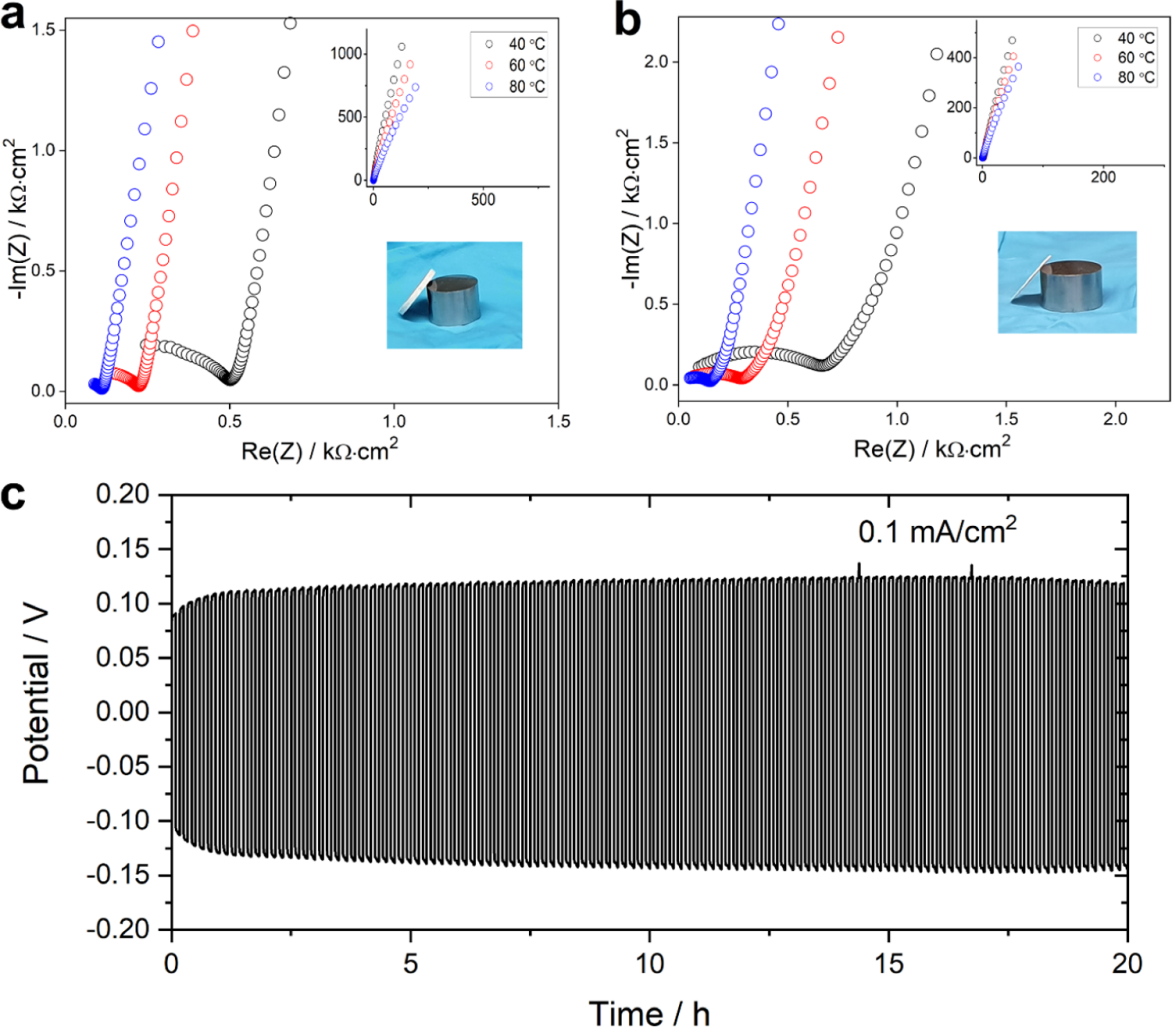
Impedance spectra collected from (a) thick GMF-free single-phase LPS and (b) thin LPS/GMF pellets at different temperatures employing C-coated Al foils as electrodes. The main figures magnify the high-frequency parts of the spectra; full spectra are attached as insets. (c) Galvanostatic cycling of a symmetric Li/LPS-GMF/Li cell at 0.1 mA cm^–2^ at room temperature.

To study the effect of the GMF scaffold on impedance, pellets were prepared of LPS/GMF (18 mg total mass) and single-phase, GMF-free LPS (100 mg total mass) using a 10 mm die set under a 250 MPa pressure. These resulted in pellets of thicknesses ∼160 and 900 μm, respectively. The scale of the die was selected to mimic the ∼10 mm diameter bulk LPS featured in previous literature reports.^26,27^ The resulting impedance data, collected from both pellets at different temperatures, are shown in Figure 2a,b. The set of temperatures shown in Figure 2 (40, 60, 80 °C) was selected to imitate the heating often employed to attain fast-ion conductivity in LPS phases synthesized by the liquid-phase route.^26,28^ The bulk ion conductivity of LPS/GMF at room temperature (22 °C) was 1.47 × 10^–5^ S cm^–1^, which increases by an order of magnitude to 1.30 × 10^–4^ S cm^–1^ at 80 °C. The bulk ion conductivity of LPS was found to be higher, 1.37 × 10^–4^ S cm^–1^ at room temperature (22 °C) and 1.70 × 10^–3^ S cm^–1^ at 80 °C, due to the absence of inert GMF. Furthermore, the activation energy for Li-ion transport in LPS/GMF and LPS (Figure S4) was found by an Arrhenius analysis as 0.38 and 0.42 eV, respectively. Figure 2 displays comparable resistances from the two GMF-free and LPS/GMF pellets, illustrating that a reduction to one-fifth of the thickness can be achieved with a negligible effect on the total resistance of the battery. Interestingly, Randau et al.^8^ have recently shown that reducing the thickness of the LPS layer from 425 to 210 μm in an all-solid-state battery can lead to a significant increase of the energy density from 72 to 172 Wh L^–1^.

To assess the compatibility of the LPS/GMF membranes with the Li-metal anode, a symmetrical lithium cell was examined by galvanostatic cycling at 0.1 mA cm^–2^. The stable cycle performance of the symmetric cell (Figure 2c) observed indicates good compatibility of LPS/GMF membranes with Li-metal anodes, reminiscent of the parent LPS material. The slight increase in resistance with time is consistent with galvanostatic cycling data of Li/LPS/Li symmetric cells previously reported,^8,16^ wherein LPS is expected to react with lithium metal to generate an interphase consisting of Li_2_S and Li_3_P.^29^ Continued galvanostatic cycling showed the stability of the symmetric cell up to approximately 50 h, although a failure of the cell was observed afterward (Figure S5). This was not observed for bulk LPS, which is currently under further investigation.

**Figure 3:**
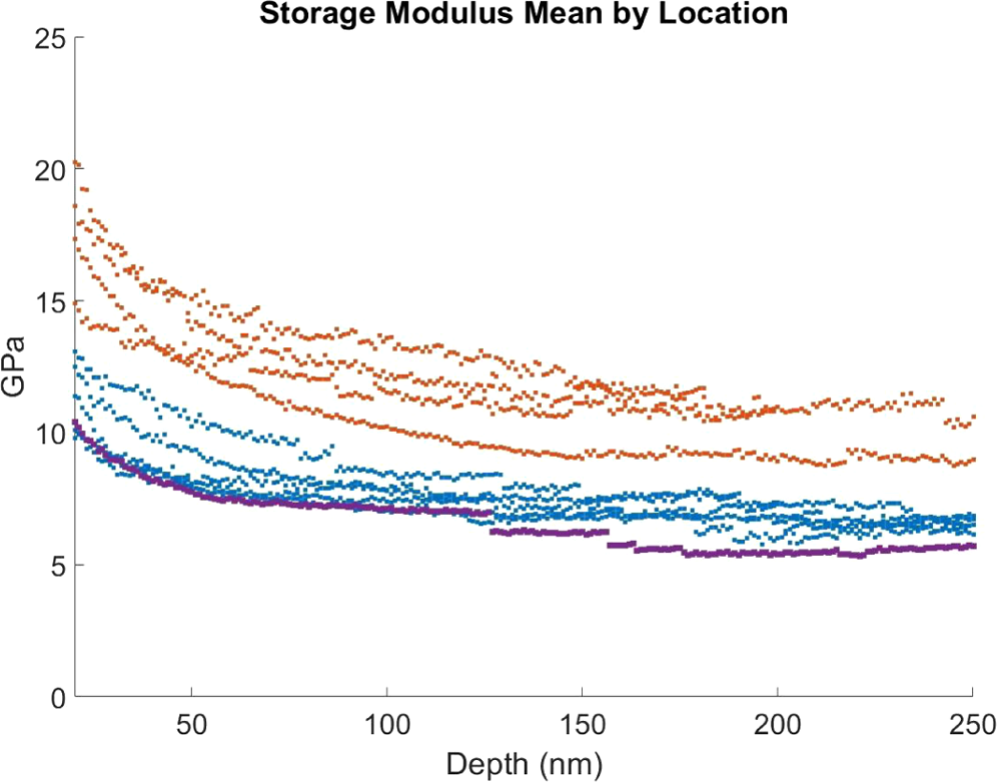
Illustration of the indentation modulus with depth for different locations with LPS in blue, LPS/GMF in orange, and a low-density region of LPS/GMF in purple.

A major benefit of the GMF scaffold is seen in the improvement in mechanical properties (see Tables 1–7; SI). In situ nanoindentation has allowed for testing of these air-sensitive materials without the need for mineral oil or other techniques that can affect the mechanical response.^30^ The indentation modulus value calculated for LPS of 7.0 ± 0.5 GPa agrees with previously reported results, and the increase observed to 11.7 ± 1.1 GPa for LPS/GMF is significant (Figure 3). As with the work of Baranowski et al.,^30^ an effect of local density can be observed with lower density areas giving lower elastic moduli. To consider the material as a bulk, therefore avoiding local density, pellets of LPS and LPS/GMF were cut into strips and tested using the in situ nanoindenter as cantilevers. This allows for the measurement of flexural modulus and strength, which are more applicable to the ease of handling and fragility seen when using these materials to construct all-solid-state batteries. The result from these tests shows that the flexural modulus is more similar than from nanoindentation (LPS 7.2 ± 0.5 GPa and LPS/GMF 8.5 ± 1 GPa). This comes from the fact that, in bending, the material is tested both in compression (as in nanoindentation) and in tension. Unpicking these two contributions shows that the tensile modulus for the LPS is slightly greater than that for LPS/GMF. This effect is common in disordered short fiber composites if the interface between the matrix and fiber is poor, meaning the mechanical response is only that of the LPS but it has a reduced volume compared to a material without fibers.^31^ The ∼0.25 mm thick cantilevers were tested to maximum bending stresses of 15–20 MPa, which the LPS/GMF endured with no observable fracturing, while an LPS cantilever failed at 17.5 MPa. Measuring the energy to fracture, as the area under the load–displacement curve (4.2 J), and fracture surface size, directly via SEM images of the cross section, a fracture toughness of 0.17 ± 0.07 MPa m^1/2^ can be calculated. This can be taken therefore as the lower limit for LPS and is a tenth of the fracture toughness of a glass ceramic.^32^ The resistance to fracture of the LPS/GMF will be due to the scaffold of material throughout even if, as observed in the tension case, the interface is weak; there are still a number of mechanisms by which the fibers support the material and increase the fracture strength.^33^ It is beyond the scope of the current study to identify the responsible mechanism(s).

**Figure 4:**
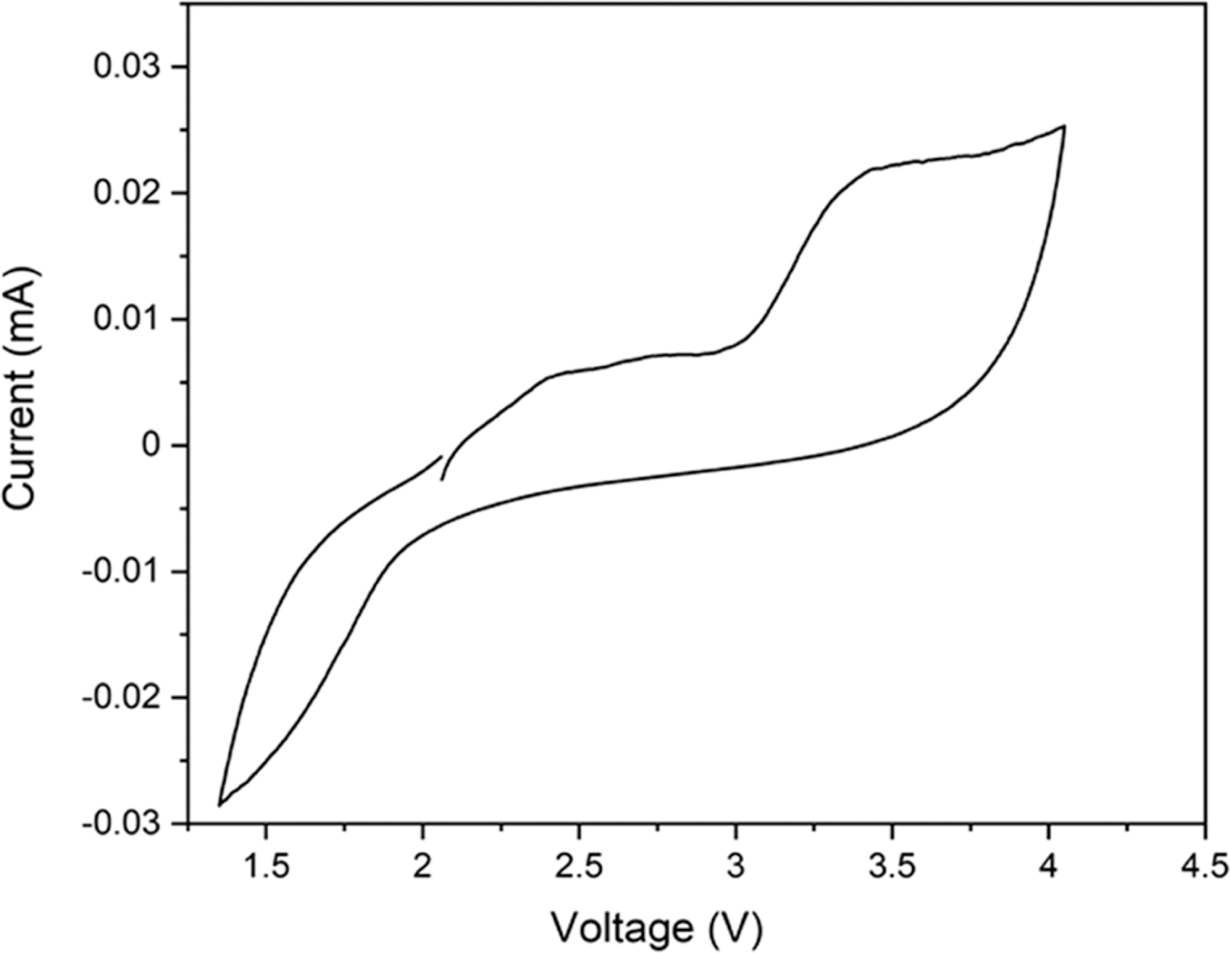
Cyclic voltammetry scan of an asymmetric Li/LPS-GMF/C cell at room temperature with a scan rate of 0.1 mV s^–1^.

In order to confirm the electrochemical stability window of LPS, cyclic voltammetry was performed on an asymmetric Li/LPS-GMF/C cell in the region 1.4–4.0 V with a scan rate of 0.1 mV s^–1^, which is displayed in Figure 4. The measurement indicates good stability of LPS up to 3 V, with significant oxidative decomposition processes occurring above 3 V, agreeing well with a previous study by Zeier et al.^34^ As such, to test the synthesized LPS/GMF membranes in full solid-state-battery configuration, Li metal and nanostructured LiTi_2_(PO_4_)_3_ (LTP) were applied as the anode and cathode materials, respectively. LTP was selected as the cathode active material since its redox potential lies within the expected electrochemical stability window of the LPS electrolyte.^35,36^

Cycling tests of the Li/LPS-GMF/LTP all-solid-state cell were performed in the potential range of 1.5–3.0 V, with measurements taken at 70 °C to enhance ionic conductivity.^16,36^ Initially, we found that solid-state batteries based on LTP/LPS/C cathode composites were not stable, likely due to the electrochemical reactivity of LPS in the presence of carbon additives (see Figure S6 for the typical cycle performance of these architectures). Bulk, LPS-free, LTP/C cathodes also showed reduced capacity due to insufficient Li-ion conduction pathways in the cathode (the ionic conductivity of LTP is an order of magnitude lower than LPS).^36^ To alleviate these issues, thin cathode layers of carbon-composited nanostructured LTP were employed.^37^ Nanostructured LTP/C composites were synthesized according to previous reports,^22^ with the material dispersed on a thin Al foil and pressed with the LPS/GMF membrane. Lithium, prepressed on thin Ni foil, was then attached to the opposite side of the LPS/GMF membrane to construct the battery.

**Figure 5:**
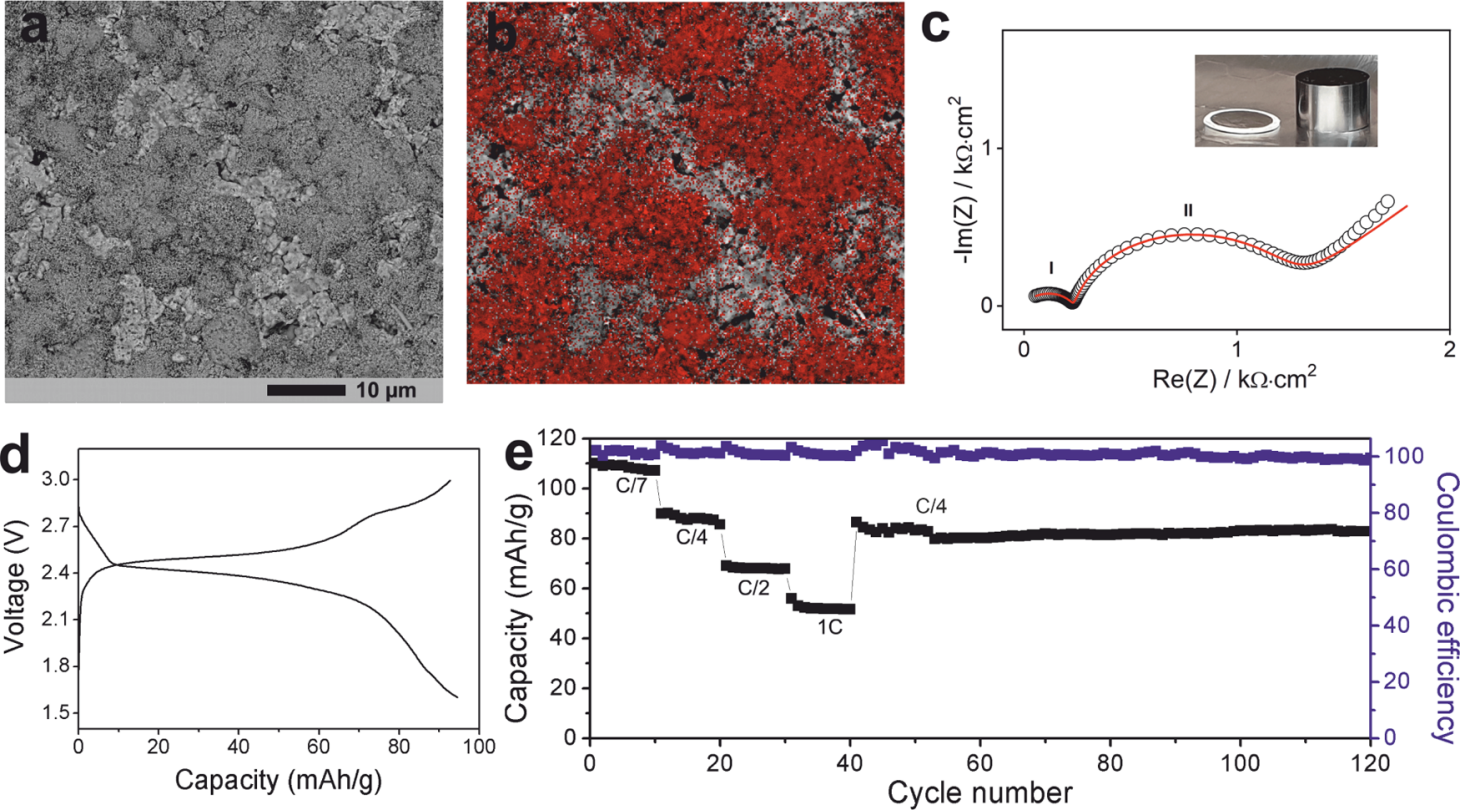
(a) SEM and (b) EDX mapping of the cathode side of the Li/LPS-GMF/LTP solid-state cell. In the EDX map, the surface Ti distribution is highlighted in red, illustrating the adhesion of the LTP phase to the LPS/GMF membrane. (c) Impedance plot of the full cell after assembling (a photograph of the cell is shown in the inset) at 70 °C. The impedance data was fitted using the equivalent circuit [*R*_1_*Q*_1_][*R*_2_*Q*_2_]*W*, where [*RQ*] is a constant phase element in parallel to a resistance element, and *W* is a Warburg diffusion element. (d) Typical discharge/charge profile of the all-solid-state cell at 70 °C at C/4. (e) Specific capacity as a function of cycle number for the solid-state cell at different charge/discharge rates at 70 °C (1C is equivalent to 138 mA g^–1^).

This process produced good-quality interfaces evidenced by impedance spectroscopy and postcycling SEM imaging. Figure 5 shows SEM images and EDX mapping of the cathode side of the cathode/electrolyte interface in this battery, where good adhesion of the LTP to the electrolyte surface is indicated by the EDX mapping of Ti (Figure 5b). Impedance data collected from the full battery prior to cycling at 70 °C is presented in Figure 5c. The impedance represented by the first semicircle in the high-frequency range is in excellent agreement with the resistance of the LPS/GMF membrane (∼0.3–0.25 kΩ cm^2^ at 70 °C; see Figure 2), suggesting that the second semicircle in the intermediate-frequency range (∼1 kΩ cm^2^) corresponds to charge-transfer resistances at the Li/LPS and LPS/LTP interfaces.^27,38^ In the low-frequency region, a Warburg impedance may be assigned to the chemical diffusion of Li^+^ in the cathode LTP particles.^27^ The cell was successfully cycled at 70 °C, showing a typical discharge/charge profile of LTP in the voltage range of 1.5–3.0 V (Figure 5d) and a capacity of up to 110 mAh g^–1^ at a C/7 rate, where 1C is equivalent to 138 mA g^–1^ (Figure 5e). The battery retained a capacity of ∼90 mAh g^–1^ (C/4 rate) after 120 cycles at different current densities (Figure 5e).

These results indicate the successful integration of LPS/GMF membranes in all-solid-state batteries employing Li anodes, which is expected to enable the construction of high-energy-density cells, providing that suitable cathode composites are employed. This system indicates good compatibility between LPS and LTP through low interfacial resistances and excellent capacity retention of the solid-state battery.

## Conclusions

We have demonstrated that the incorporation of GMF during the liquid-phase synthesis of solid LPS yields bulk-type GMF-reinforced solid electrolytes, which can be easily processed as thin membranes by cold pressing. This approach preserves the scalability of the solution-based approach technique and is amenable to all-liquid-phase reactions since GMF is chemically inert and withstands the temperature ranges often employed in liquid-phase synthesis (up to 300 °C). We show that LPS/GMF membranes are compatible with Li-metal anodes, as indicated by the successful operation of an all-solid-state cell comprising a Li/LPS-GMF/LTP configuration. These GMF-reinforced solid electrolyte membranes deliver improved mechanical properties while retaining good conductivity, allowing for the reduction of the mass of the bulk material. In the current report, a one-fifth reduction in the solid electrolyte layer compared with standard solid-state approaches was demonstrated. We anticipate that further design of composite cathodes with greater loading of active material will afford higher-energy-density solid-state batteries.

## Supporting Information Available

Impedance data for different wt % GMF composites, additional XRD and SEM data, Arrhenius analysis of impedance data, extended cycling data, and tabulated mechanical strength data are provided in the supplemental information (PDF)
