# Supplementary material for: Liquid-Phase Approach to Glass-Microfiber-Reinforced Sulfide Solid Electrolytes for All-Solid-State Batteries
Source: ACS Appl Mater Interfaces. 2023 Jul 19;15(30):36512–8. doi: 10.1021/acsami.3c01383 (PMC10401568; doi:10.1021/acsami.3c01383)
Supplement: Supplementary file 1 — am3c01383_si_001.pdf [file am3c01383_si_001.pdf]

SUPPORTING INFORMATION

A liquid-phase approach to glass-microfiber-reinforced sulfide solid-electrolytes for all-solid-state batteries

Hany El-Shinawi, [▪#†] *Ed Darnbrough,* [‡#]*, Johann Perera,* [‡#] *Innes McClelland,*[▪#] *David E. J. Armstrong,* [‡#] *Edmund J. Cussen,* [▪#] *Serena A. Cussen* [▪#]*

▪ Department of Materials Science and Engineering, University of Sheffield, Mappin St, Sheffield City Centre, Sheffield S1 3JD, United Kingdom

[†]Chemistry Department, Faculty of Science, Mansoura University, Mansoura 35516, Egypt

‡ Department of Materials, University of Oxford, Parks Road, Oxford OX1 3PH, United Kingdom

The Faraday Institution, Quad One, Harwell Science and Innovation Campus, OX11 0RA, UK

*Corresponding Author: Serena A. Cussen, s.cussen@sheffield.ac.uk

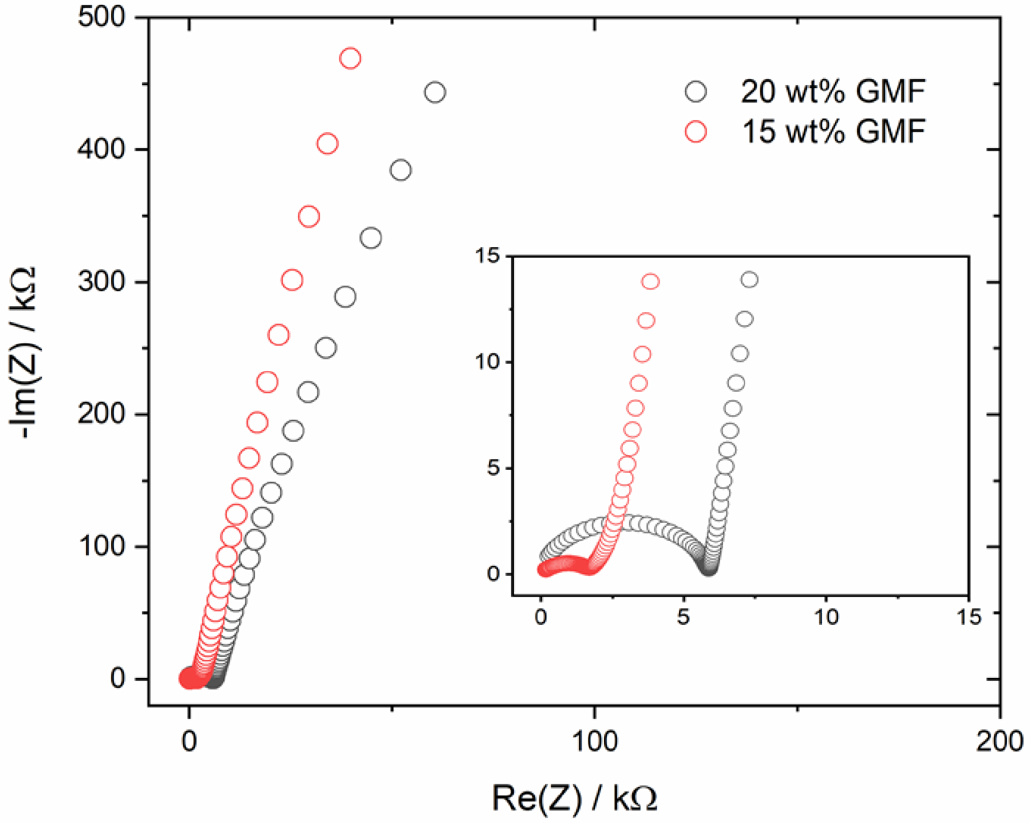

Figure S1. Impedance spectra collected from thin LPS/GMF pellets at different compositions, both measured at room temperature. Pellets were prepared using 20 mg of material pressed at 125 MPa using 10 mm dies. The inset show the higher frequency region at higher magnification. The 15wt% GMF composition was preferred for further studies due to its improved conductivity.

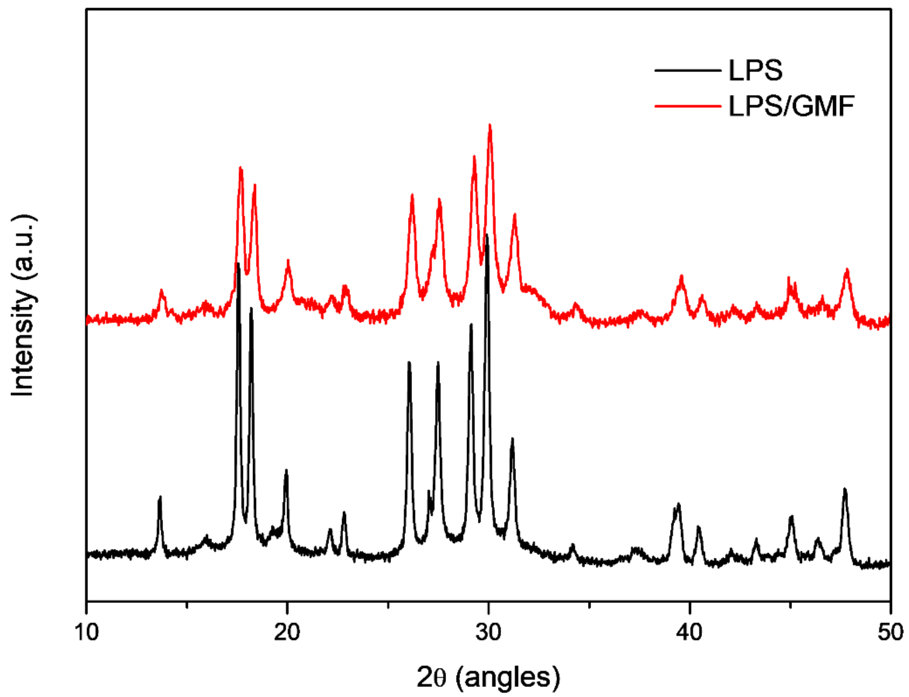

Figure S2. XRD patterns collected from as-synthesized LPS and LPS/GMF.

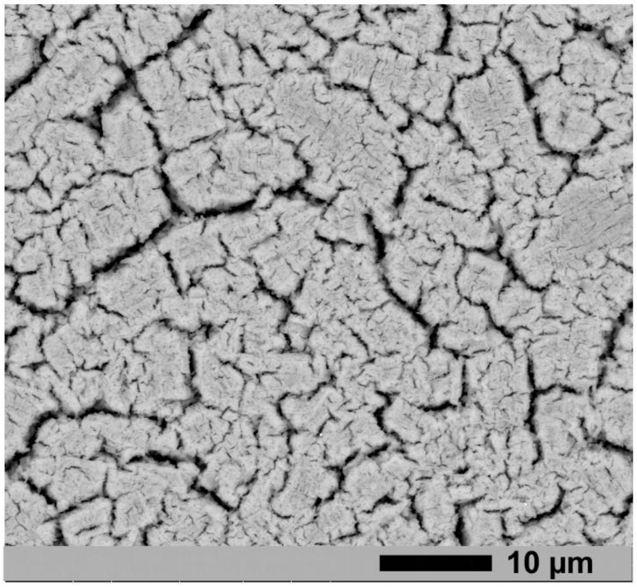

Figure S3. SEM image of GMF-free LPS, pressed at 125 MPa using 10 mm dies.

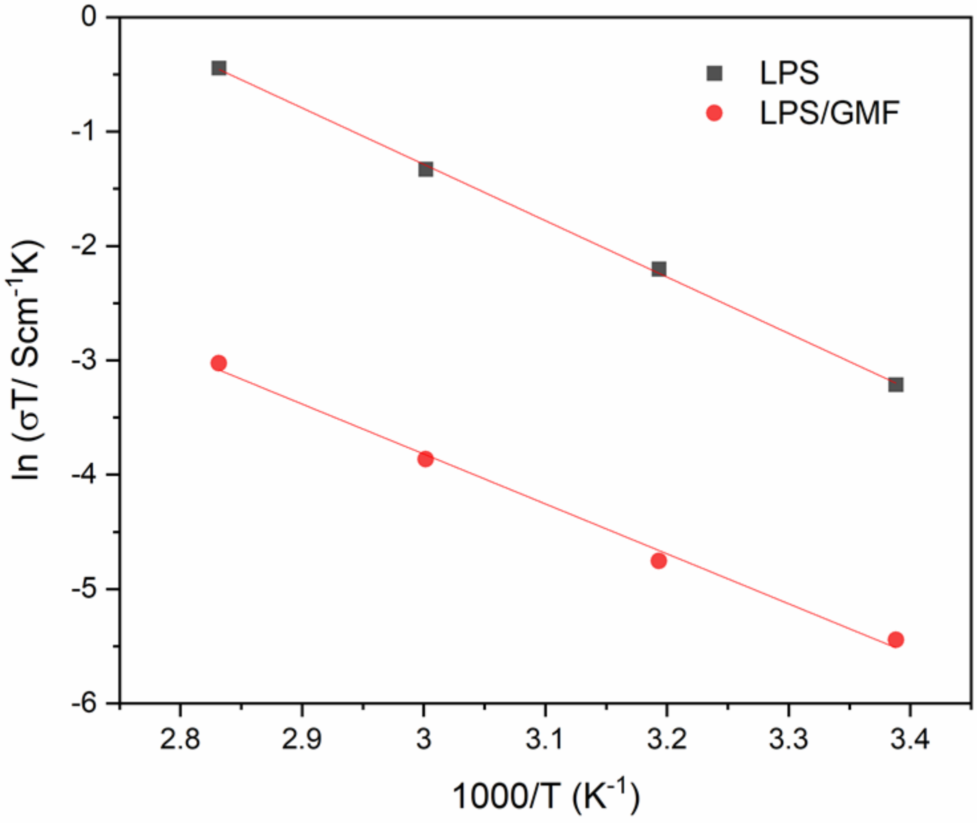

Figure S4. Arrhenius plots found by variable temperature impedance measurements of both LPS and LPS/GMF pellets. The activation energy of LPS and LPS/GMF was found to be 0.42 and 0.38 eV, respectively.

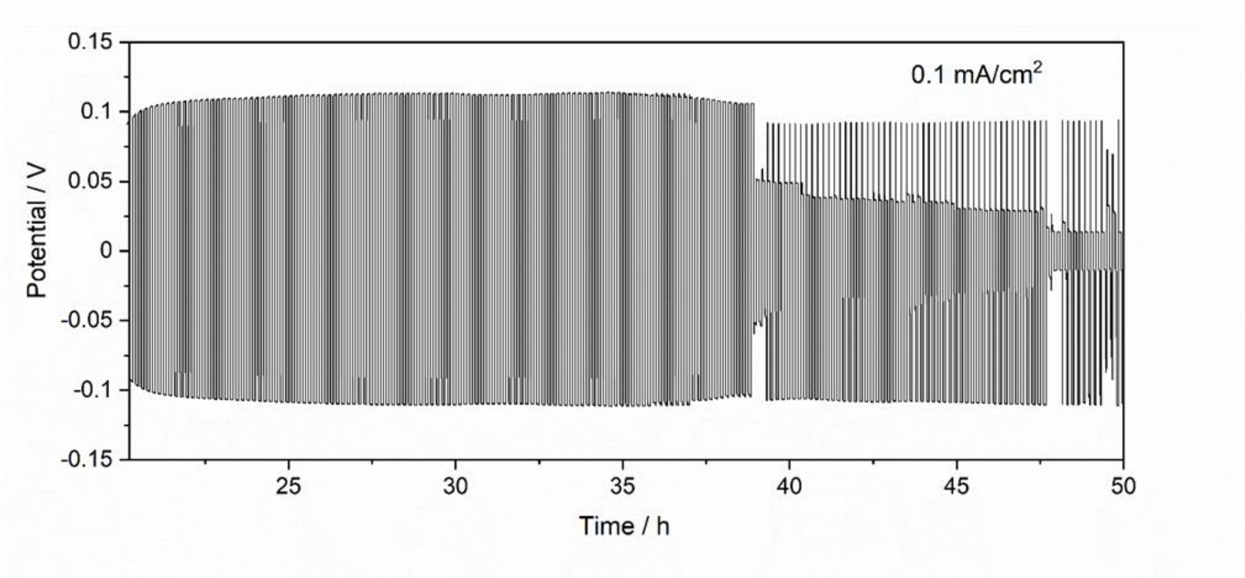

Figure S5. Galvanostatic cycling of a symmetric Li/LPS-GMF/Li cell at 0.1 mA cm^{-2} at room temperature over an extended time region.

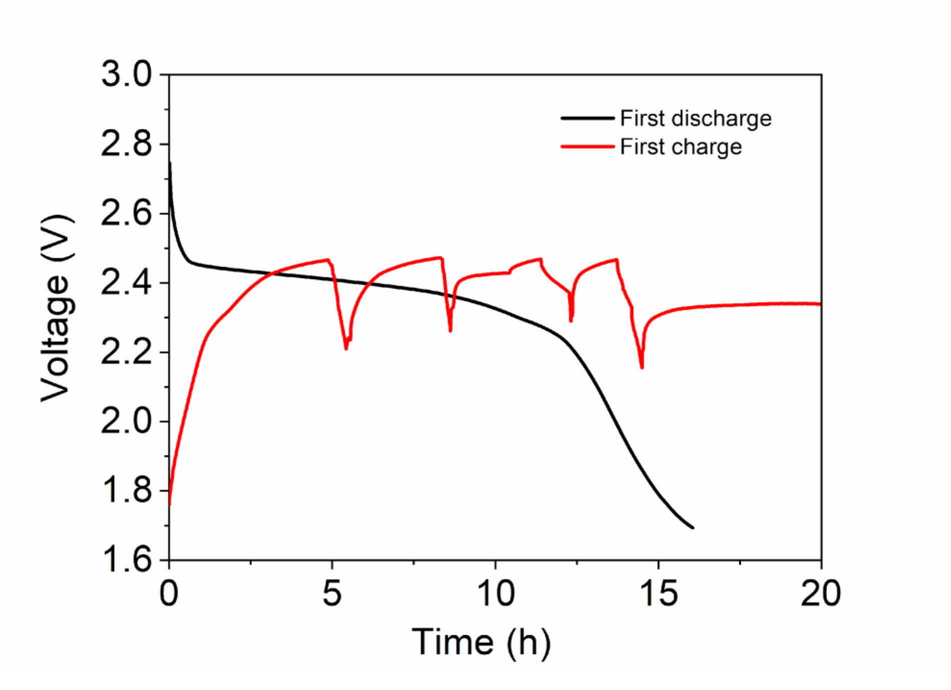

Figure S6. First discharge and charge of a Li/LPS-GMF/LTP-LPS-C solid-state cell at C/15 rate. The cell employs Li metal as anode, LPS/GMF membrane as the solid electrolyte, and a mixture of LTP/LPS/C (6:3:1) as the cathode. The failure of the battery, at the charge step, is likely due to a redox activity of LPS in the presence of carbon additives.

Table S1. Showing indentation Modulus by location in sample without GMF.

Location	Mode	Variance	Standard Error	[all in GPa]
1	7.4083e+00	5.4789e+00	6.4920e-01	
2	6.8012e+00	2.8020e+00	5.0470e-01	
3	7.2402e+00	1.6572e+00	4.2911e-01	
4	7.0194e+00	1.7866e+00	4.2268e-01	
5	6.3906e+00	4.1214e+00	6.4198e-01	
Mean	6.9719e+00	3.1692e+00	5.2953e-01	

Table S2. Showing indentation Modulus by location in sample with GMF.

Location	Mode	Variance	Standard Error	[all in GPa]
1	1.2118e+01	7.6299e+00	1.0440e+00	
2	1.3083e+01	1.5254e+01	1.3019e+00	
3	1.1837e+01	1.8029e+01	1.2802e+00	
4	9.8293e+00	1.2374e+01	1.1726e+00	
Mean	1.1717e+01	1.3322e+01	1.1997e+00	

Table S3. Showing Hardness by location in sample without GMF.

Location	Mode	Variance	Standard Error [all in GPa]
1	4.6716e-01	3.0905e-02	4.8758e-02
2	3.8910e-01	2.9659e-02	5.1926e-02
3	4.9430e-01	9.1761e-03	3.1931e-02
4	4.4882e-01	1.3433e-02	3.6651e-02
5	4.4140e-01	1.9235e-02	4.3857e-02
Mean	4.4816e-01	2.0482e-02	4.2625e-02

Table S4. Showing Hardness by location in sample with GMF.

Location	Mode	Variance	Standard Error [all in GPa]
1	1.1112e+00	7.3322e-02	1.0235e-01
2	1.2476e+00	9.0475e-02	1.0026e-01
3	6.4850e-01	6.2871e-02	7.5602e-02
4	7.4050e-01	7.0104e-02	8.8257e-02
Mean	9.3693e-01	7.4193e-02	9.1617e-02

Table S5. Showing indentation Modulus and Hardness in low density GMF region of sample with GMF.

**Indentation Modulus**:	5.1385e+00	3.3396e+00	5.5100e-01
**Hardness**:	2.9567e-01	1.6270e-02	3.8458e-02

Table S6. Showing results of cantilever bend test of sample without GMF.

Width 2.8181e+00 mm Height 2.5192e-01 mm

**Length (mm)**	**Modulus from Unloading (Pa)**
1.1136e+00	6.8270e+09
1.3330e+00	7.9242e+09
1.5524e+00	7.5315e+09
1.7485e+00	7.0720e+09
1.9680e+00	7.8727e+09
1.9680e+00	6.2872e+09
2.1825e+00	6.9608e+09

Mean (Pa) 7.2108e+09 standard deviation 5.9611e+08

Calculated tensile modulus (Pa) 7.4642e+09 8.2015e+08

Bending Stress at Fracture : 1.7472e+07 Pa

Energy to fracture : 4.2502e+00 J

Stress Intensity Factor : 1.7517e+05 Pa m1/2 (+- 7.1226e+04)

Table S7. Showing results of cantilever bend test of sample with GMF.

Width 1.8482e+00 mm Height 3.1796e-01mm

**Length (mm)**	**Modulus from Unloading (Pa)**
9.1819e-01	9.8246e+09
1.1909e+00	9.3073e+09
1.5217e+00	9.9907e+09
2.1556e+00	9.1350e+09
3.0167e+00	8.0068e+09
3.9409e+00	7.4163e+09
3.9409e+00	8.2940e+09
5.4965e+00	8.0486e+09
5.4965e+00	7.0324e+09

Mean (Pa) 8.5617e+09 standard deviation 1.0497e+09

Calculated tensile modulus (Pa) 6.5272e+09   1.0428e+09

Max bending stress : 2.0449e+07 Pa
